# Cytomegalovirus Infection Impairs Immune Responses and Accentuates T-cell Pool Changes Observed in Mice with Aging

**DOI:** 10.1371/journal.ppat.1002849

**Published:** 2012-08-16

**Authors:** Luka Cicin-Sain, James D. Brien, Jennifer L. Uhrlaub, Anja Drabig, Thomas F. Marandu, Janko Nikolich-Zugich

**Affiliations:** 1 Department of Vaccinology and Applied Microbiology, Helmholtz Center for Infection Research, Braunschweig, Germany; 2 Vaccine and Gene Therapy Institute and the Oregon National Primate Research Center, Oregon Health and Science University, Portland, Oregon, United States of America; 3 Department of Microbiology, Washington University School of Medicine, St. Louis, Missouri, United States of America; 4 Department of Immunobiology and the Arizona Center on Aging, University of Arizona College of Medicine, Tucson, Arizona, United States of America; McMaster University, Canada

## Abstract

Prominent immune alterations associated with aging include the loss of naïve T-cell numbers, diversity and function. While genetic contributors and mechanistic details in the aging process have been addressed in multiple studies, the role of environmental agents in immune aging remains incompletely understood. From the standpoint of environmental infectious agents, latent cytomegalovirus (CMV) infection has been associated with an immune risk profile in the elderly humans, yet the cause-effect relationship of this association remains unclear. Here we present direct experimental evidence that mouse CMV (MCMV) infection results in select T-cell subset changes associated with immune aging, namely the increase of relative and absolute counts of CD8 T-cells in the blood, with a decreased representation of the naïve and the increased representation of the effector memory blood CD8 T-cells. Moreover, MCMV infection resulted in significantly weaker CD8 responses to superinfection with Influenza, Human Herpes Virus I or West-Nile-Virus, even 16 months following MCMV infection. These irreversible losses in T-cell function could not be observed in uninfected or in vaccinia virus-infected controls and were not due to the immune-evasive action of MCMV genes. Rather, the CD8 activation in draining lymph nodes upon viral challenge was decreased in MCMV infected mice and the immune response correlated directly to the frequency of the naïve and inversely to that of the effector cells in the blood CD8 pool. Therefore, latent MCMV infection resulted in pronounced changes of the T-cell compartment consistent with impaired naïve T-cell function.

## Author Summary

The cytomegalovirus (CMV) is a widespread virus of the herpesvirus family, which latently infects the majority of the adult human population worldwide. While CMV causes severe disease in AIDS patients, in recipients of organ transplants, or when infection occurs during pregnancy, this virus is considered apathogenic for the general population. Several reports indicated that CMV infection may be associated with poor immune function, and even survival, in older adults, yet it remained unclear whether CMV infection may have the potential to impair the immune function. Here we test this possibility in a mouse model of CMV (MCMV) infection. We found that mice carrying latent MCMV exhibit significant changes in CD8^+^ lymphocytes, which are crucial in the recognition of, and protection against viruses. These changes were similar to changes observed in aging and resulted in poor cytotoxic T-lymphocyte response to infection with unrelated viruses, such as the West Nile virus or the influenza virus. Our study provides the first experimental demonstration that latent CMV infection may impair the immune defense of the host.

## Introduction

Cytomegalovirus (CMV) is a ubiquitous herpesvirus, latently persisting in the majority of the adult human population worldwide [1]. While CMV may induce severe disease in immunocompromised patients, it is generally considered apathogenic for the adult immunocompetent host. Broadly targeted CMV-specific T-cells dominate the memory compartment of exposed subjects [2], and CD8 T-cells specific for an immunodominant epitope are more frequent in people belonging to older age groups [3], peaking in the very old [4]–[5]. Epidemiologic studies in healthy elderly volunteers have identified a correlation of CMV seropositivity and the immune risk profile (IRP), a condition characterized by the accumulation of CD28^−^ CD8 T-cells, poor proliferative responses to polyclonal stimulation, inverted CD4/CD8 T-cell ratios [6]–[7], and, in some cohorts, decreased life expectancy of the host [6], [8]–[10]. Others have shown that CMV seropositivity correlates to significant telomere shortening in the T-cell pool [11], poor response to influenza vaccination [12] or poor immunity to a co-resident EBV infection [13]. Therefore, it has been proposed that the massive commitment of the memory T-cell pool to the control of CMV infection may contribute to an accelerated onset of immune senescence [14], where the filling of the immunological space with CMV-specific T-cells may constrict the T-cell receptor (TCR) repertoire. More recent studies raised some doubt on these findings (for a review see the recent Report from the Second Cytomegalovirus and Immunosenescence Workshop [15]), arguing that not all aging human populations exhibit IRP [16], yet others showed a correlation between CMV seropositivity and frailty in the elderly [9], [17] or increased mortality [9]. However, while the clinical studies could show the association of CMV infection and parameters associated with immune senescence, they cannot elucidate the cause-effect relationship between these phenomena. Therefore, it was not clear if CMV infection causally contributes to immune senescence or if immune senescence or predisposition for other immune alterations results in an increase of susceptibility to CMV infection.

The cause-effect relationship between CMV and immune senescence may be defined by an experimental approach. Such a study would require the comparison of experimentally infected hosts to uninfected ones over the course of their lifetime, which is only feasible in animal models of infection. Due to its strict species specificity, *in vivo* experimental models of CMV infection and immunity rely on the infection of animals with orthologous CMV viruses. We decided to use the mouse model of infection because lifelong experiments are feasible, CMV-uninfected control hosts are readily available, and the mouse CMV (MCMV) model is well characterized [18], recapitulating the essential features of CMV infection and immunity. CD62L^lo^ (effector memory - EM) CD8 T-cells with MCMV specificity accumulate once the primary infection has been cleared and viral latency has been established [19]. This accretion of memory T-cells has been termed memory inflation (MI), and shown to proceed continuously over the host's lifetime [20], resulting in an accumulation of CMV-specific CD8 cells exhibiting activated CD28^−^CD27^−^CD122^−^ phenotypes [21]. Therefore, the mouse model reflects the accumulation of differentiated CMV-specific cells observed in human CMV infection [3]. MI likely reflects an ongoing recruitment of MCMV-specific naïve and central memory cells [22]–[23], because the MCMV specific EM cells are unable to proliferate in response to Ag upon adoptive transfer [22]–[23], which is in line with the proliferative hyporesponsiveness of the accumulated CMV-specific CD8 cells in elderly humans [11], [24], or in rhesus CMV (RhCMV) specific CD8 T-cells in old or adult rhesus monkeys [25]. It remained unclear whether the changes of the T-cell compartment upon CMV infection are irreversible and progressive and whether CMV affects the overall functionality of the T-cell pool.

We show here evidence that MCMV infection results in profound changes that affect the entire CD8 pool. The changes included a relative and absolute increase of CD8 T cell counts, a persistent increase of its EM fraction and a reduced representation of the naïve CD8 cells, leading to a distorted TCR repertoire diversity. Moreover, we show that CD8 responses to superinfection with West-Nile virus (WNV), influenza or Herpes Simplex virus are diminished. These changes occurred also upon infection with an MCMV recombinant lacking all known immune evasive genes, arguing that they were not caused by direct CMV interference with the immune system. On the other hand, the strength of the response to WNV correlated inversely to the representation of the naïve pool and directly to that of the EM CD8 pool, thus linking the MCMV-induced changes in phenotype and homeostasis to the functional response to superinfection. An analysis of lymphoid compartments showed that MCMV infection resulted in an overall increase in the size of all CD8 compartment in lymph nodes, yet simultaneously in poor mobilization of CD8 cells to the draining LN upon a viral challenge.

## Results

### MCMV infection results in a decrease of CD4/CD8 ratio due to an absolute increase of CD8 T-cells

**Figure 1:**
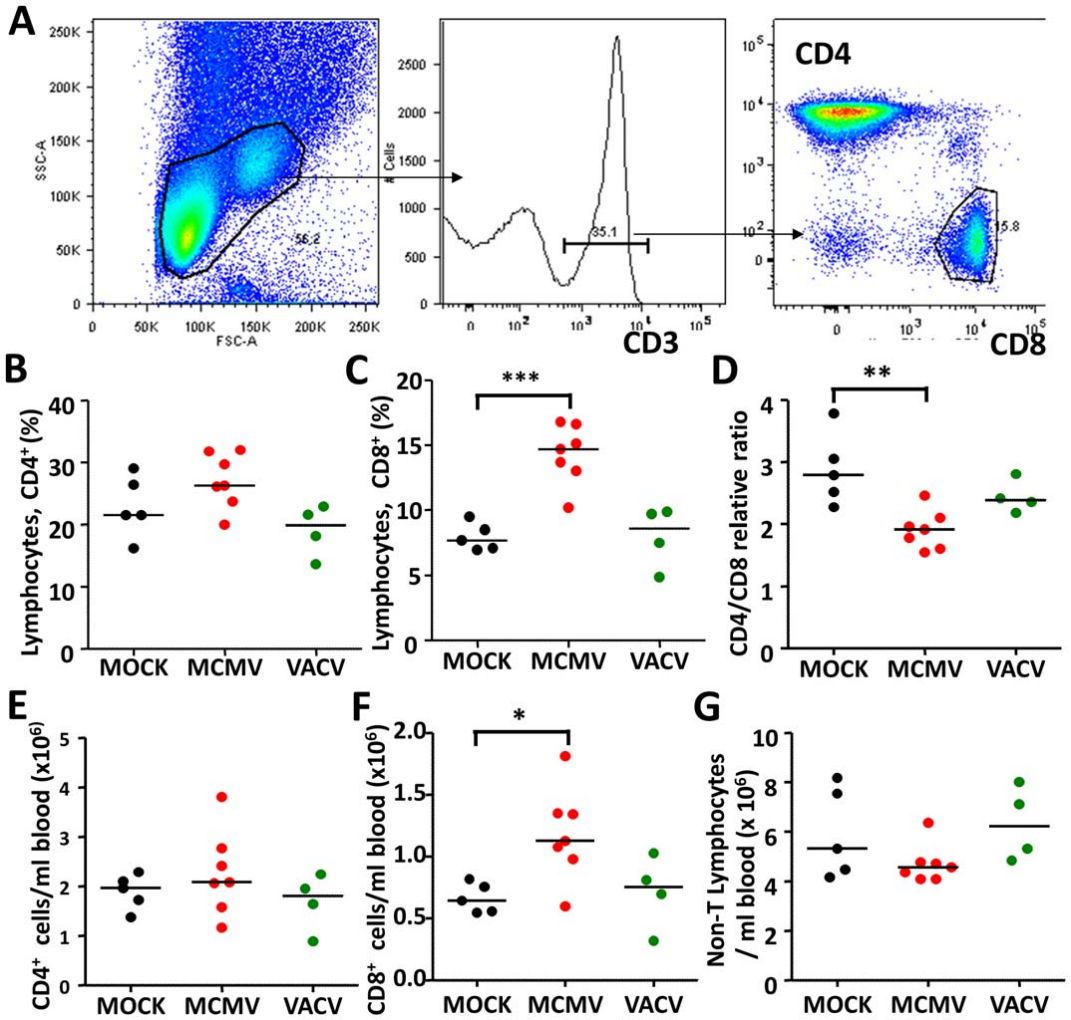
Cytomegalovirus infection irreversibly perturbs the T-cell pool. Blood leukocytes of mice infected with 10^5^ PFU of MCMV, 10^6^ PFU of VACV-IE1 or injected with PBS were analyzed at 9 months post infection by flow cytometry and representative gating is shown (A). Pecentages of CD4^+^ T-cells (B) or CD8^+^ T-cells (C) in the lymphocyte pool were divided in individual mice to calculate the CD4/CD8 ratio (D). Cells were in parallel acquired in a cell counter, and the lymphocyte count was multiplied with the fraction of CD4 or CD8 cells in the lymphocyte population, to calculate the absolute number of CD4 T-cells (E) or CD8 T-cells (F) per ml of blood. The percentage of cells out of either gate was multiplied with the lymphocyte count to establish the absolute count of non-T lymphocytes (G). Symbols denote values from individual mice, horizontal lines show medians. Significant (p<0.05) group differences upon ANOVA and Dunnet Post analysis are indicated, where * - p<0.05; ** - p<0.01; *** - p<0.001.

IRP has been described as the inversion of the CD4/CD8 ratio in the peripheral blood due to an accumulation of CD8 T-cells. [10]. We therefore compared the peripheral blood of 15 month old mice experimentally infected for 9 months with MCMV, to uninfected littermate mice (MOCK). To define if any change would be specific for MCMV infection, or a phenomenon that may be observed in any other infection, we included in our experiment a control group of mice infected with a recombinant vaccinia virus (VACV) expressing the immunodominant MCMV gene IE-1 (VACV-ie1). Flow cytometric analysis (Fig. 1A) revealed that the CD4 cell pool was not significantly altered (Fig. 1B), whereas the percentage of CD8 cells increased significantly in the MCMV-infected group (Fig. 1C), resulting in a decrease of the CD4/CD8 ratio (Fig. 1D). While the ratio was not inverted, as described in some cohorts of elderly humans with IRP [10], the observed changes tilted the CD4/CD8 balance towards a relative increase of CD8 cells in the T-cell pool.

To understand whether the ratio was altered due to an increase in the count of CD8, a decrease of CD4 T-cells, or combined changes that may have affected the non-T lymphocyte compartment, we defined their absolute counts per ml of blood. We did not observe changes in the CD4 compartment (Fig. 1E), but found a significant increase in the CD8 count (Fig. 1F). Cells that did not belong to either the CD4 or the CD8 pool were defined as non-T cells, and their number was also not altered (Fig. 1G). Therefore, latent CMV infection resulted in an absolute and a relative increase of the peripheral CD8 pool, which decreased the CD4/CD8 ratio.

### MCMV infection results in persistent activation of a large fraction of the CD8 pool

**Figure 2:**
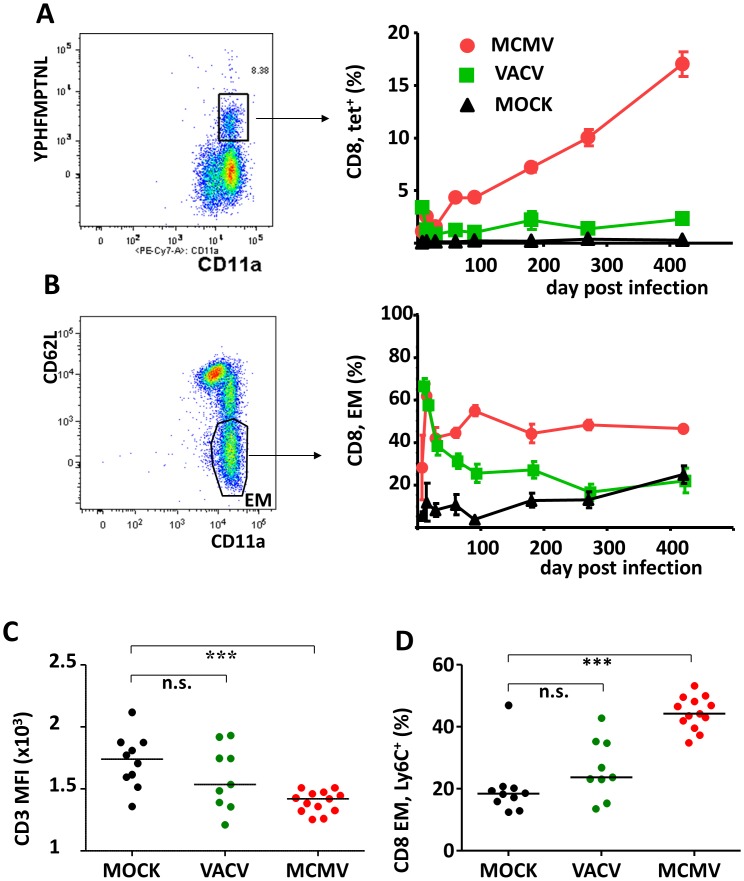
Cytomegalovirus infection irreversibly perturbs the CD8 T-cell pool. (A, B) 6 months old mice were infected with 10^5^ PFU of MCMV or 10^6^ PFU of VACV-IE1 and compared to untreated controls. Blood leukocytes were stained for CD3, CD4, CD8, CD11a, CD62L and YPHFMPTNL-L(d) tetramers and analyzed by flow cytometry. (A) CD8 T-cells were gated on tetramer^+^ CD11a^+^ gate as indicated in the representative plot on the left and group means (+/− SD) at 7, 14 28, 60, 90 180, 270 and 420 days post infection are connected. (B) The same cells as in panel A were gated on a CD62L^−^CD11a^+^ gate to identify EM cells (plot on the left) and group means (+/− SD) for indicated days are connected. (C) At 9 months post infection, we gated CD8^+^CD4^−^ lymphocytes and compared the surface TCR expression, defined as the mean fluorescence index (MFI) of CD3, in the MCMV, VACV and mock infected mice. Symbols indicate individual mice, horizontal lines are medians. n.s. – p>0.05; *** - p<0.001 according to Bonferroni statistical comparison. (D) At 9 months post infection, the frequency of activated (Ly6C^+^) cells in the CD8 EM lymphocytes of MCMV, VACV or mock infected mice was compared by Bonferroni statistical comparison. Symbols indicate individual mice, horizontal lines are medians. n.s. – p>0.05; *** - p<0.001.

Since the most prominent changes were observed in the CD8 pool, we focused on this subset. Memory inflation (MI) is an ongoing accumulation of MCMV-specific CD8 T-cells with EM (CD62L^lo^) phenotypes in the peripheral pool [19]–[20]. To understand the effects of MI on the EM CD8 T-cell pool, we compared the kinetics of antigen specific responses (Fig. 2A) to that of the frequency of EM cells (Fig. 2B). We followed mice infected with MCMV, VACV, or MOCK infected littermates. As expected, in MOCK controls we observed no increase in the antigen specific cells and a modest accumulation of EM cells starting at 300 days and consistent with the age-related changes in the T-cell pool [26]. In VACV-infected control mice, we observed an initial strong response to infection, both in terms of IE-1 specific responses and the increase in the total EM pool, followed by contraction of responses upon virus clearance. Low levels of IE1-specific cells could be detected throughout 14 months post infection, while the EM fraction decreased to levels observed in uninfected controls by 9 months post infection, again consistent with the age-related changes in the immune system.

In MCMV infection the response showed a distinctly different pattern: there was an expansion, brief contraction and then an ongoing accumulation (memory inflation, MI) of IE-1-specific cells in the peripheral pool (Fig. 2A), consistent with previous observations, [19]–[20]. The total EM pool showed a significant expansion immediately upon infection, and then after a brief dip, returned to a plateau and remained consistently expanded throughout the period of observation, taking up nearly half of the total CD8 pool (Fig. 2B). The IE-1 epitope is only one of the epitopes present in MCMV, and the vast response to primary infection, seen as the acute increase in the total EM cell pool, likely represented the sum of antiviral response to different epitopes. The subsequent persistence of large numbers of EM-cells may have likewise reflected persistent Ag stimulation, in which case one would expect that such EM cells may exhibit signs of recent Ag contact. We examined the activation status of the CD8 pool at a late time point upon infection (9 months), and have observed a decrease of TCR expression on CD8 T-cells as evidenced by the reduction of CD3 (Fig. 2C) or TCR β chain expression (data not shown). Similarly, MCMV infection resulted in the increase of the activation marker Ly6C in the CD62L^lo^ pool (Fig. 2D, see also Fig. S1), arguing for repeated activation of this subset. Overall, the MCMV infection resulted in a permanent increase of EM cells bearing an activated phenotype.

### The persistence of the EM expansion in MCMV infection is independent of the aging process

**Figure 3:**
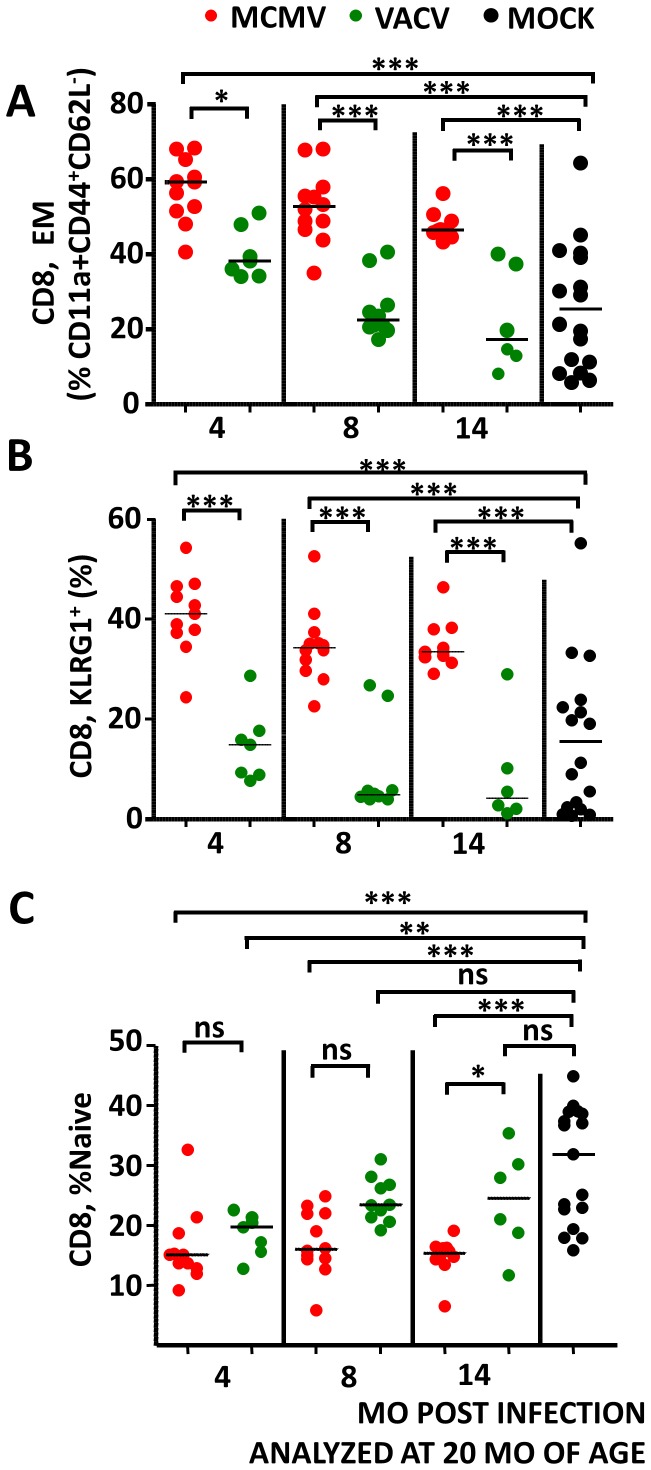
Long-term maintenance of effector and decrease of naïve CD8 subsets upon MCMV infection. Mouse littermates were infected at 6, 12 or 16 months of age with MCMV (red symbols) or VACV-IE1 (green symbols) and compared at 20 months of age (4, 8 or 14 months post infection, as indicated below x axes) to uninfected controls (black symbols). (A) CD8^+^ cells were gated on CD11a^+^CD44^+^, then on a CD62L^−^ gate, and frequencies of CD11a^+^CD44^+^CD62L^−^ cells in the CD8 pool were calculated. Each symbol represents a mouse, horizontal lines indicate medians. (B) CD8^+^ cells from mice shown in panel A were gated on a KLRG1^+^ gate, and their frequency in individual mice is shown. Each symbol represents a mouse, horizontal lines indicate medians. Significance was assessed by ANOVA followed by Bonferroni post-analysis for indicated columns (* - p<0.05, *** - p<0.001). (C) Naïve cells were defined by progressive gating on a CD11a^−^CD44^−^, and then on a CD127^+^CD62L^+^ gate (see Fig. S2). Each symbol represents a mouse, horizontal lines indicate medians. Significance was assessed by ANOVA followed by Bonferroni post-analysis for indicated columns (ns – p>0.05, * - p<0.05, ** - p<0.01, *** - p<0.001).

To differentiate between effects upon the EM compartment caused by natural aging processes and those driven by persistent MCMV infection, we compared littermate mice infected at 6, 12 or 16 months of age once they reached 20 months of age (14, 8 and 4 months post infection, respectively). The relative size of the EM pool exhibited strong variability with age in mock-infected controls (Fig. 3A), consistent with age-related changes (see Fig. 2B). In VACV-infected control mice, we observed that the fraction of EM cells in the CD8 pool was elevated at 4, lower at 8 and the lowest at 14 months post infection, consistent with the decrease of this fraction over time (see also Fig. 2B). In MCMV infection, this decrease was notably slower and the EM pool remained significantly larger than in mock-infected controls at all time points (Fig. 3A). The increase in the fraction of KLRG1+ cells in virus infected mice has been described in several models of infection [27]–[28], and in human CMV specific CD8 T-cells [29] and was shown to remain elevated only in cells responding to persistent viruses, but not to CD8 cells reacting against acute viruses [29]. The persistence of activated T-cells upon MCMV infection was confirmed by the pattern of persistent surface expression of KLRG1, a marker expressed on the surface of activated cells. While VACV infection resulted in a moderate and transient increase of the KLRG1^+^ subset of CD8 cells, their fraction was persistently elevated in MCMV infected mice, (Fig. 3B).

### MCMV infection irreversibly diminishes the representation of naïve cells in the CD8 pool

It was conceivable that the persistence of the EM subset affected the size of the naïve T-cell pool. Thus, we determined the fraction of the CD44^−^CD11a^−^CD62L^+^CD127^+^ pool (representative gating in Fig. S2) at 20 months of age in mice infected at 6, 12 or 16 months of age with MCMV or VACV and compared it to the frequency of naïve CD8 cells in mock-infected mice. We observed that both infections resulted in lower frequencies of naïve cells at 4 months post infection, yet this decrease was persistent only in MCMV infection (Fig. 3C). Moreover, at 14 months post infection VACV infected mice showed significantly higher fractions of naive cells than MCMV infected ones. Therefore, only the MCMV infection resulted in an irreversible decrease of the fraction of naïve cells, and that occurred regardless of the age at which infection occurred, indicating that CMV infection may readjust the homeostatic balance of the CD8 T-cell pool.

### MCMV infection alters the TCR repertoire

**Figure 4:**
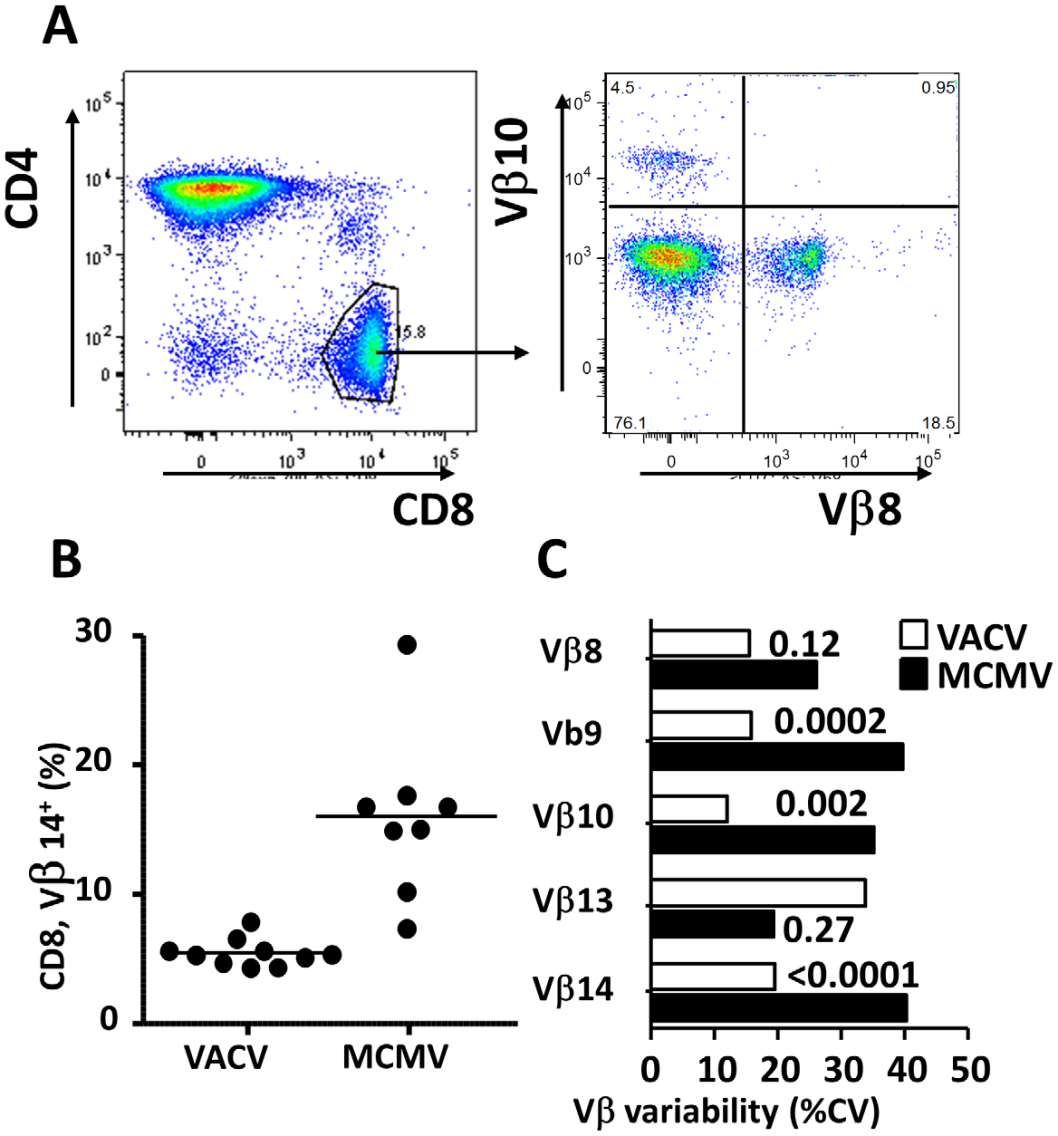
Vβ family analysis of CD8 pools upon MCMV infection. (A,B,C) BALB/cxDBA/2 F1 Mice infected at 6 months of age with MCMV or VACV-IE1 as in Fig. 1A and 1B were bled at 14 months following infection and analyzed for frequency of Vβ8, Vβ9, Vβ10, Vβ13 and Vβ14 populations. Representative gating for Vβ 8 and Vβ10 is shown (A). Frequencies of CD8 cells belonging to Vβ families were analyzed in individual mice and a representative analysis is shown for Vβ14 (B), where each mouse is displayed by a symbol, and group medians by horizontal lines. The cohort variability of Vβ population frequencies were defined in groups of MCMV or VACV infected mice (C), and are indicated as SD on the x axis. Variance F-tests were performed to identify differences in group variabilities and p values are indicated in the chart.

It has been shown that human CMV infection results in significant changes in the pool sizes of TCR families, defined by the variable ® chain (Vβ) expression [30]. Moreover, while young mice exhibit remarkably consistent frequencies of T-cells with defined Vβ, aging results in increased variability of TCR Vβ pool sizes [31]. We compared the variability of the various Vβ fractions of CD8 cells in MCMV and in VACV-infected mice at 14 months post-infection and 20 months of age (representative blot in Figure 4A, representative comparison of Vβ14 frequencies Fig. 4B). The coefficients of variability of Vβ8, Vβ9, Vβ10, Vβ13, and Vβ14 subsets were compared by the F-test of variances and three out of five tested Vβ families (Vβ9, Vβ10 and Vβ14) showed significantly higher variability in MCMV infected mice than in VACV infection (Fig. 4C), while the other two showed no significance. These results argued for a significant increase in the variability of the size of Vβ TCR families and hence a significant change of the TCR repertoire in the CD8 pool, concomitant to the increase of EM and the reduction of naïve cell representation in the CD8 pool of MCMV infected mice.

### CD8 population changes in lymph nodes and spleen of MCMV infected mice differ from the blood

**Figure 5:**
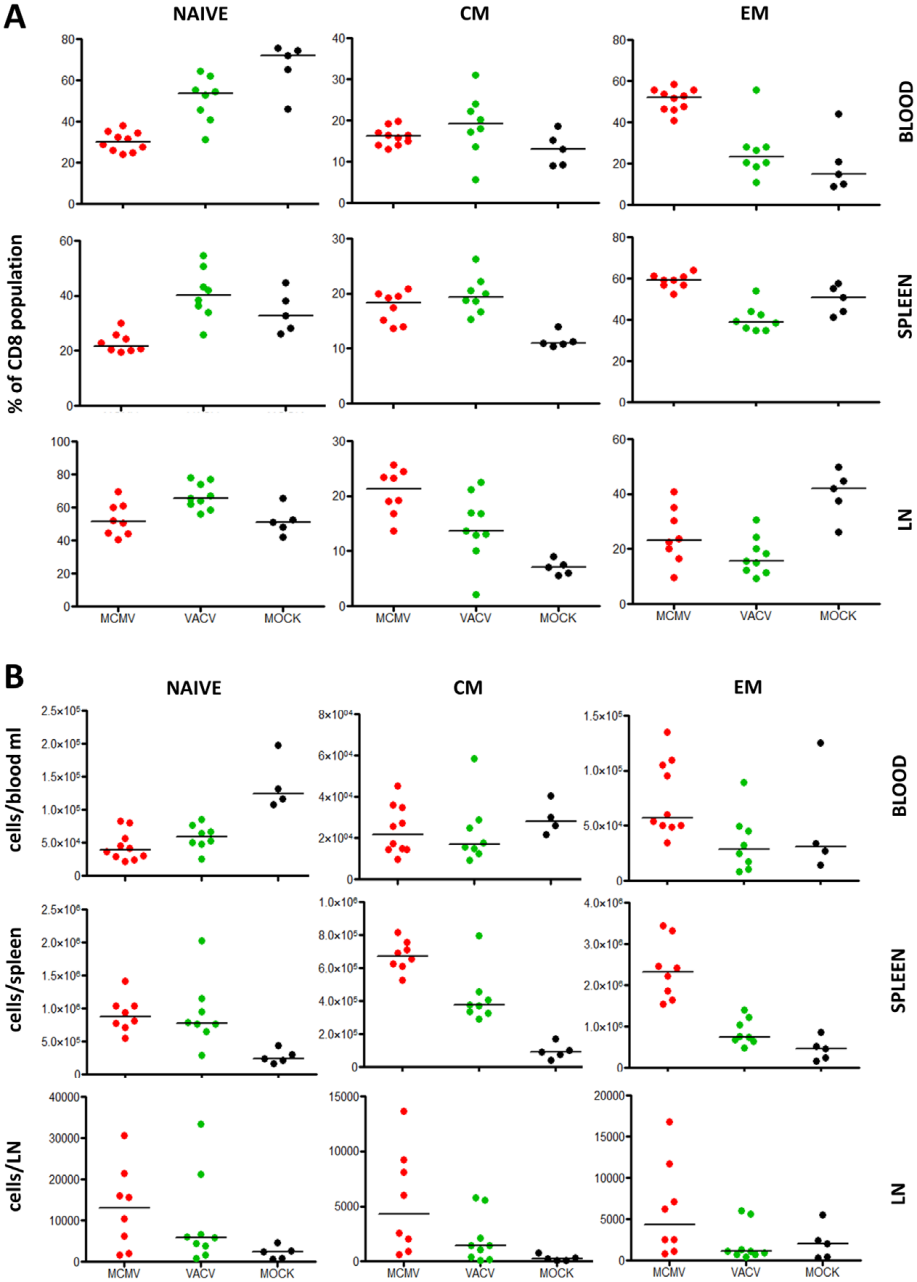
Relative and absolute counts of naïve, CM and EM cells in blood, spleen and LN of MCMV, VACV or MOCK-infected mice. 3 month-old BALB/c mice were injected with 2×10^5^ PFU of MCMV, 10^6^ PFU of VACV or 200 µl PBS (mock infected). 6 months later blood, spleen and inguinal LN CD8^+^ cells were gated on CD11a, CD44, CD62L and CD27 (for a representative gating strategy, see Fig. S3) to define (A) the relative representation and (B) the absolute count of naïve, CM and EM subsets in each compartment. Each dot represents data from a single mouse, horizontal lines show medians.

Changes in the blood compartment of MCMV infected mice matched the population changes observed in HCMV-infected human subjects, arguing that the mouse model may offer translational insights about the effect of CMV infection. Recently, Remmerswaal et al. showed that lymph nodes in humans do not contain effector memory T cells specific for CMV [32]. To examine the effect of the controlled mouse CMV infection on the lymphocyte populations in lymphoid organs, including the spleen or lymph nodes (LN), we compared the percentages of naïve, CM and EM cells in the spleen and inguinal LN of MCMV, VACV and MOCK infected mice at 6 months post infection (Fig. 5A). The spleen population distribution partially matched those observed in the blood, where MCMV resulted in decreased naïve and increased EM percentages, although the difference was significant only between MCMV and VACV infected mice (Fig. 5A). The LN showed a distinctly different pattern, with an elevated proportion of CM in MCMV and VACV infected mice and, surprisingly, the highest proportion of EM cells in MOCK-infected controls, while the naïve pool contained a similar proportion of naïve cells in the LN of all mice (Fig. 5A). Therefore, we concluded that the decrease of naïve and increase of EM cell representation in MCMV infection was specific for the blood, and to a lesser extent for the spleen compartment, but not for the LN.

### Absolute changes in CD8 populations match the relative changes in the blood, but not in the spleen and LN

To evaluate whether the change in the fractions of T-cells were a result of the loss of naïve cells, increase in EM, or a combination of both, we quantified the absolute counts of cell subsets in all compartments tested in the previous section. We observed an obvious absolute reduction of naïve in the blood of MCMV and VACV infected mice (Fig. 5B), but also an increase in the size of the EM pool in the blood of MCMV infected mice, partially reflecting the changes in proportions (Fig. 5A). On the other hand, in spleen and LN we observed an absolute increase of all CD8 subsets in MCMV and VACV infected mice over the MOCK controls, with the increase more pronounced in the MCMV that in the VACV group (Fig. 5B). This was in line with our observation that spleen and LN were enlarged in infected mice as compared to the mock controls and that the mean total CD8 number in the LN was more than threefold increased in MCMV infected mice compared to the mock-infected controls (not shown). Hence, while we observed a loss of naïve cells in the blood compartment of MCMV infected mice, which was not the case in the secondary lymphoid organs of the same mice.

### MCMV infection decreases CD8 responses to superinfecting viruses

**Figure 6:**
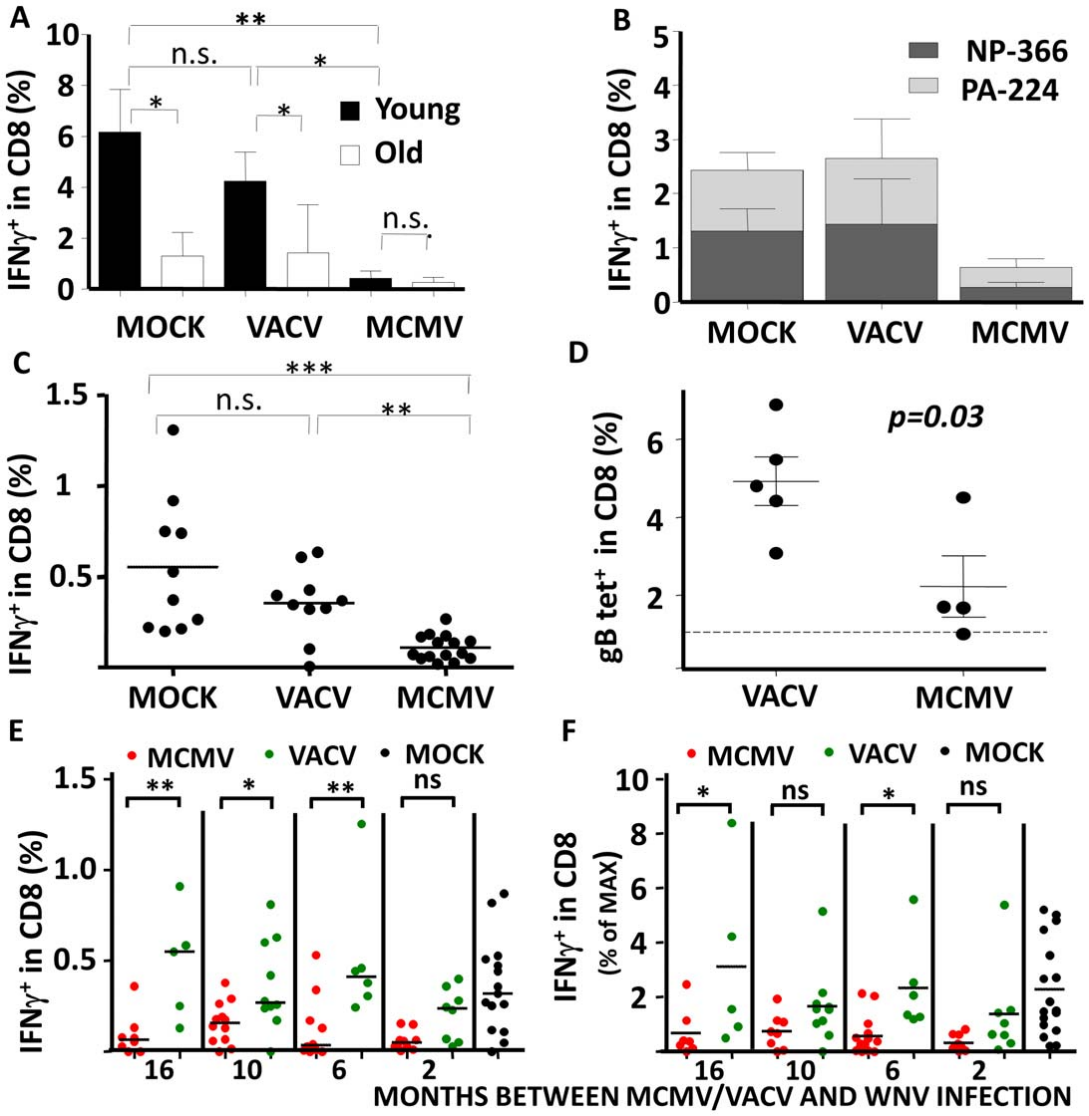
CD8 T-cell responses to superinfection upon MCMV infection. (A) groups of 3 (young) and 12 (old) month old mice were primed with 10^5^ PFU of MCMV, 10^6^ PFU of VACV-WR or PBS (MOCK), and challenged 5 months later with 300 EID of influenza virus, PR/8 strain, intranasally (i.n.). 7 days post challenge blood lymphocytes were tested by ICCS for IFNγ responses to a 9 h in vitro stimulation with the NP^366^ peptide in the presence of BrefeldinA. Mean IFNγ responses in animal groups (n = 5/group)+SD is shown on the y axis. (B) 12 month-old mice were primed as indicated and challenged 5 months later with Influenza. The frequency of peptide specific cells in the blood CD8 pool was defined by peptide restimulation and ICCS. Group average (+SD) values for either peptide are indicated on the y axis. (C) Mice were primed with 10^5^ PFU of MCMV or 10^6^ PFU of VACV-IE1 at 2 months of age, and i.p. challenged with 50 PFU of West Nile Virus at 8 months of age. 7 days later, blood lymphocytes were in vitro stimulated with a pool of two immunodominant H^2d^ restricted WNV peptides and analyzed by ICCS. The frequency of IFNγ expressing T-cells in the CD8 pool of individual mice is displayed on the y axis. Horizontal lines indicate medians. (D) C57BL6/DBA2 F1 mice were primed with MCMV or VACV-IE1 at 3 months of age, challenged with HSV-1 at 8 months of age and frequencies of HSV-1 specific responses were determined 7 months later by pMHC tetramer staining and FCM. Each mouse is indicated with a symbol, horizontal lines are means, the dashed line shows the detection threshold. The p value for Mann-Whitney analysis is shown. (E, F) Mice were primed with MCMV or VACV-IE1 at 6, 12, 16 or 20 months of age, and assayed for responses to WNV challenge at 22 months of age (16, 10, 6 or 2 months post prime, as indicated below axis) using the protocol as in panel C. Each mouse is indicated with a symbol, horizontal lines are means. (E) % of CD8 cells responding to peptide stimulation in ICCS. (F) Leukocytes were in parallel assayed by polyclonal stimulation with anti-CD3 antibodies, and the peptide specific response was normalized to the CD3 (Max) response. Values in panels A, C, E and F were compared by 1-way ANOVA followed by Bonferroni comparison of individual columns, and significance is indicated (n.s. - p>0.05, *- p<0.05, ** - p<0.01, *** - p<0.001).

The CMV-induced perturbations in the representation and pool size of naïve and memory cells in multiple compartments. The altered CD8 T-cell repertoire could have affected the functional response of the naïve pool to heterologous microbial challenge. To test this assumption, we challenged 129Sv6 mice with viruses unrelated to MCMV or VACV and monitored T-cell responses by means of peptide in vitro restimulation. Five months following MCMV, VACV or mock infection, we challenged adult (8-month old) and aging (17-month old) mice with influenza virus. CD8 responses to the influenza NP_366_ peptide were significantly weaker in aging mice, than in adult ones (Fig. 6A), but only in the mock-infected and in the VACV infected controls. Mice carrying latent MCMV infection showed weak responses to influenza both in the adult and in the aging group. More importantly, CD8 responses were significantly weaker in MCMV-infected than in control-infected mice, arguing that latent MCMV infection results in poor T-cell responses upon superinfection with influenza. Weak CD8 responses to another flu-derived immunodominant peptide (PA_224_) showed that this loss of response was not limited to the NP_366_ peptide in old (Fig. 6B) nor in adult mice (data not shown).

To exclude the possibility that the suppression of naive-cell responses to novel antigen depends on the mouse strain and challenge virus, we challenged BALB/cxDBA/2 F_1_ mice with WNV. We observed significantly weaker CD8 responses to the WNV peptides only in mice latently infected with MCMV, (Fig. 6C). In a third unrelated experiment we used DBA/2xC57BL/6 F_1_ mice and Herpes simplex virus type 1 (HSV-1) as the challenge virus. Mice were infected with MCMV or VACV at 3 months of age, challenged 5 months later with HSV-1, and monitored for the frequency of CD8 cells specific for the immunodominant, K^b^-restricted, HSV-1 peptide SSIEFARL. In MCMV infected mice we observed a trend towards weaker responses at early time post infection (not shown) and significantly reduced long-term CD8 memory at 7 months post challenge (Fig. 6D). To understand if the function of naïve cells could be restored over very long periods of time, we compared mice infected at 6, 12, 16 or 20 months of age with MCMV or VACV to mock-infected controls, by challenging them with WNV at 22 months of age (18, 10, 6 and 2 months post MCMV infection, respectively). MCMV infection resulted in a reduction of CD8 responses to WNV peptides in all MCMV groups (Two-way ANOVA relative to mock controls p<0.0001), and WNV-specific responses were higher in all VACV-infected groups (Fig. 6E), with the possible exception of mice infected with VACV at 20 months of age. Interestingly, this phenomenon seemed specific for MCMV infection but not another chronic Herpesvirus infection, as old mice persistently infected with HSV-1 were indistinguishable from uninfected littermates in terms of CD8 responses to WNV challenge (Fig. S4).

Blood cells from same mice shown in Fig. 6E were stimulated with anti-CD3 antibodies for 6 h to control for the possibility that CMV may have induced general hyporesponsiveness of CD8 T cells, yet the responses were essentially identical in all mice groups (Fig. S5). More importantly, the fraction of WNV-peptide responding cells was normalized to the percentage of anti-CD3 responsive cells, to obtain the percentage of maximum response. Again, we could observe the weakest response in MCMV infected mice, arguing that the poor response was specific to the CD8 ability to recognize WNV peptides, and not a result of overall immune suppression in MCMV infected mice (Fig. 6F). In conclusion, our results strongly argued that MCMV infection suppresses immediate and memory CD8 responses to superinfection with unrelated virus.

### Poor naïve CD8 responses in MCMV infected mice do not depend on known immune evasive genes, but may be explained by changes in the CD8 pool

**Figure 7:**
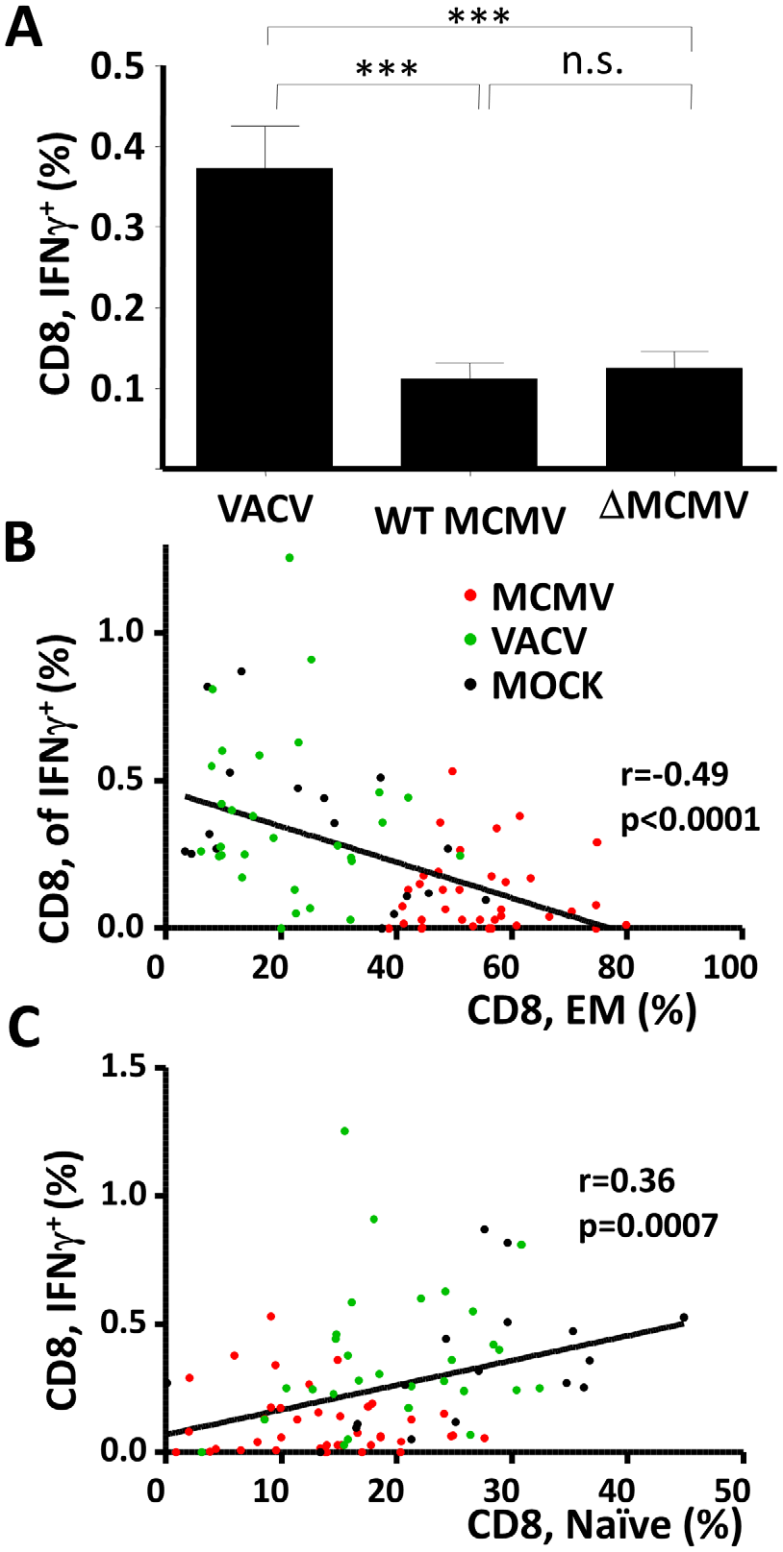
Poor CD8 response to WNV in latently infected mice is not caused by viral immune evasive genes. (A) Mice were primed with 10^5^ PFU of MCMV, 5×10^5^ PFU of ΔMCMV or 10^6^ PFU of VACV-IE1 at 6–8 months of age and challenged with 50 PFU of WNV at 22 months of age. Peptide stimulation with WNV peptides was performed as in figure 6A, and group averages+SD of IFNγ responses are indicated by histograms. Statistical comparison was performed by ANOVA followed by Bonferroni analysis of individual groups (*** p<0.0001, n.s. p>0.05). (B, C) IFNγ responses upon WNV challenge were correlated to the frequency of (B) EM (CD62L^−^) or (C) naïve (CD11a^−^CD44^−^) CD8 T-cells in individual MCMV (red dots) VACV-IE1 (green dots) or MOCK (black dots) infected mice. Trend indicates the linear correlation, Pearson r and significance (p) are indicated, where the p value indicates the probability that the trend deviates from a horizontal line.

The poor CD8 response to superinfection in MCMV infected mice could be explained by two plausible causes. One was that the changes in the CD8 pool upon MCMV infection (Fig. 1–4) resulted in their poor immune response to neoantigens. The other was that MCMV immune-evasive genes [33] suppressed the immune response to WNV or influenza superinfection. While immune evasive genes are expressed with early kinetics, and not during MCMV latency [34], superinfection may have resulted in MCMV reactivation in professional antigen-presenting cells as they matured [35]. In that case, the expression of MCMV-encoded immune evasive genes would have inhibited DC function and T-cell priming, but MCMV recombinants lacking immune evasive genes would not compromise the immune response to superinfections. Since the known MCMV genes interfering with MHC-I peptide presentation belong to the m2 or to the m145 gene families, we deleted by targeted mutagenesis all the MCMV genes belonging to these two families [36] and used the recombinant virus, termed here ΔMCMV, to define the effects of MCMV immune evasins on the CD8 responses. The CD8 responses to a WNV challenge in mice infected with ΔMCMV was suppressed just as in wild type (WT) MCMV infection (Fig. 7A). Thus, poor CD8 response to WNV challenge was unlikely due to immune evasion genes encoded by MCMV. To address the alternative scenario, in which the changes in the peripheral CD8 pool result in poor CD8 responses to superinfections, we cross correlated the fraction of naïve cells to the fraction of CD8 cells responding to the WNV challenge (Fig. 7B). We observed a significant (p = 0.0007) and direct linear correlation (r = 0.36) of naïve cell pool sizes and response intensities which argued that the size of the naïve pool has the potential to influence the response to WNV. Moreover, the size of the EM pool correlated more strictly (r = −0.49) and significantly (p<0.0001) to the WNV responses, but the correlation was inversed (Fig. 7C), arguing that the presence of large pre-existing EM pools may impair the immune response to neoantigen.

### MCMV infection results in poor mobilization of CD8 cells to draining LN

We considered next that the naïve response must initiate in the draining LN of mice upon which the naïve cells are activated to become effector cells which migrate out of the LN into the bloodstream and towards virus infected tissues. While our data showed a correlation of blood CD8 subsets to the size of the CD8 response to WNV, the changes in the blood CD8 subsets of MCMV infected mice did not reflect the situation in the LN (see fig. 5). Therefore, we reasoned that only by measuring the changes of the CD8 populations in the draining and non-draining LN upon a virus challenge we may explore if MCMV blocked the activation of CD8 cells, or their migration out of the draining LN. Intranasal influenza challenge results in lung infection upon which mediastinal LN act as draining LN, whereas inguinal or mesenteric LN are not involved in the primary responses and may be used as control LN.

**Figure 8:**
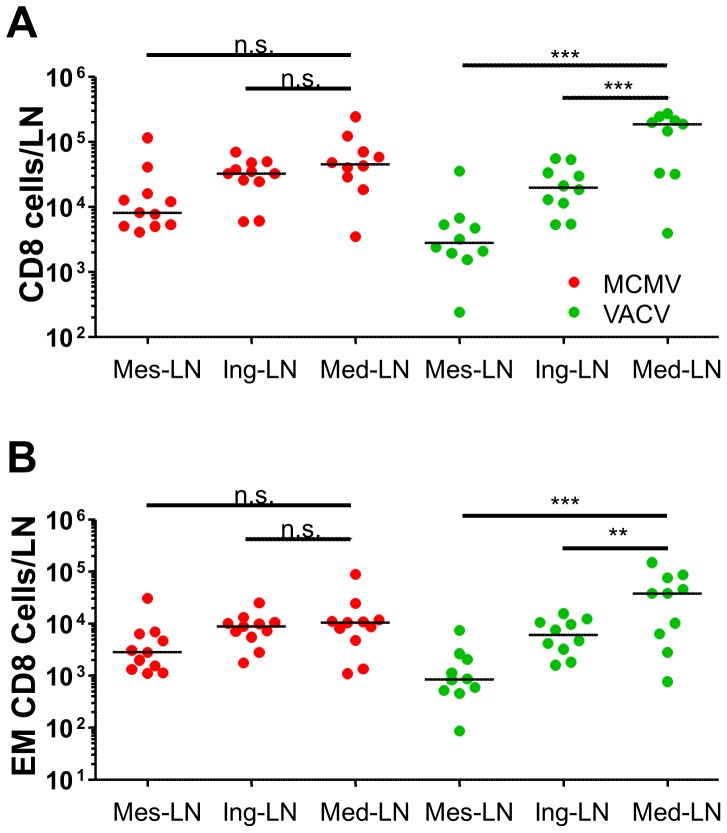
Poor mobilization of CD8 cells in draining LN of MCMV infected mice upon flu challenge. LN from 9 month old BALB/c mice infected with VACV or MCMV for six months prior to intranasal challenge with influenza virus were collected at dpi 7. (A) Cells were gated on the CD8^+^ CD4^−^ gate in an Accuri cytometer to establish absolute counts of CD8^+^ cells per mesenteric (Mes-LN), inguinal (Ing-LN) or mediastinal (Med-LN) lymphnodes. (B) CD8 cells were acquired in parallel in an LSR2 apparatus and analyzed as shown in supplementary figure 3. Dividing the CD8 cell count with the fraction of cells in the EM gate defined the absolute counts for this subset. Cell counts in individual mice are displayed, horizontal lines indicate medians. 1-way Anova followed by Bonferroni post analysis was performed to define the differences between draining and non-draining LN (***-p<0.001, **-p<0.01, *-p<0.05, n.s.-p>0.05).

Comparison of the CD8 population in the draining and control LN of mice acutely infected with influenza at 6 months post MCMV or VACV infection showed that the CD8 compartment was significantly larger in the draining LN of the VACV-infected mice, but not in those from MCMV infected mice (Fig. 8A). This argued that the recruitment and/or activation of CD8 cells in the draining LN of MCMV infected mice may be impaired. This difference was specific for the mediastinal LN of Flu-challenged mice, because a comparison of CD8 counts in MCMV and VACV infected mice in the absence of a flu challenge revealed no difference in the count of CD8 in mediastinal LN (Figure S6).

To understand which subset of CD8 cells was specifically contributing to the stronger CD8 response in the draining LN of VACV infected mice, we compared the naïve, CM and EM subset of CD8 cells in draining and control LN. We observed that draining LN presented similar numbers of naïve cells and an overall higher number of CM cells in both MCMV and VACV infected groups (data not shown), but a specific increase of the EM fraction in the draining LN that was limited to the VACV infected group (Fig. 8B). Therefore, our results argued that an infection with flu resulted in a less vigorous activation of EM cells in the draining LN of MCMV infected mice, than in those of the VACV infected controls.

## Discussion

In this study, we demonstrate that experimental CMV infection diminishes the CD8 response to heterologous virus infections. It has been shown that herpesvirus infections have the potential to improve the immune protection against bacterial infections [37] due to elevated innate immune responses. In our study we observed no positive effects of latent MCMV infection on the immune protection of mice upon WNV challenge, if anything the survival was slightly shorter and lower (L.C-S. & J.N-Z., unpublished data). During the preparation of this manuscript, we became aware of the results by the Karrer group (Mekker et al, Submitted as companion manuscript), whose results further support this interpretation – these authors found that mice latently infected with MCMV show diminished clearance of lymphoid choriomeningitis virus (LCMV) upon challenge (Mekker et al. submitted). The differences between our data and those of Barton et al [37] likely reflect differences in challenge models and perhaps more importantly, the age of experimental animals and/or the length of CMV infection and the time of testing after primary infection. While that study focused on effects occurring relatively soon after infection (45 days post infection) in young adult mice, we focused on effects occurring 5 months or later upon infection, in middle aged or old mice.

We considered the possibility that the absolute number of CD8 cells responding to WNV or flu remained unchanged, and that the lower frequencies of responding cells merely mirrored an absolute increase of memory cells due to the CMV-induced T-cell expansions. However, while CMV infection increased the size of the peripheral blood CD8 pool by a factor of 1.7 (Fig. 1C), this could not have explained the observed 10 or 12-fold differences in the frequency of flu-specific cells (see Fig. 6A), which was consistent with the absolute loss of CD8 response to LCMV challenge observed in old MCMV-infected mice (Mekker et al. submitted).

The phenotype analysis of the blood compartment argued for two main possibilities: (i) that a loss of naïve T cell numbers, and/or their TCR repertoire diversity, caused poor responses to superinfection with emerging viruses (this scenario could explain the difference between MCMV and mock-infected mice based on numerical differences, but not between MCMV and VACV infected mice, since both infections reduced the naïve CD8 count in the blood; potential TCR repertoire changes remain to be analyzed in sufficient detail); or (ii) that accumulation of large EM cell populations inhibited the response of the remaining naïve cells, which was in line with findings of others (Mekker et al. submitted). The analysis of compartments other than the blood showed an overall accumulation of CD8 cells with no loss of the naïve subset, excluding the reduced naïve cell numbers as the mechanism underlying poor responses in MCMV infected mice. Moreover, in LN of MCMV infected mice we observed a specific enrichment of the CM and not the EM fraction. This is in line with data by Snyder et al. showing that the MCMV-specific EM fraction of memory-inflated cells cannot renew on its own, but rather mirrors the proliferation of a CMV-specific subset of CM cells in the LN of infected mice [22]. Finally, and most importantly, upon an influenza challenge we observed an overall expansion of the CD8 pool in the draining LN, which was impaired in MCMV-infected mice. Detailed analysis showed that this impairment was mainly due to poor expansion and/or accumulation of effector CD8 cells. Therefore, while our results do not formally exclude the possibility that the poor immune responses were caused by other mechanisms, they suggest that latent MCMV infection specifically contributes to the poor activation of CD8 cells in the LN of mice carrying latent MCMV.

Mekker et al. also observed that young animals infected for two months with MCMV prior to challenge showed less reduction in CD8 responses to LCMV challenge than old animals, while we observed an opposite trend in adult and old mice challenged with influenza at 5 months post CMV infection. This difference likely reflects the differences in age at challenge and length of MCMV infection prior to infection, or in the choice of viruses used for challenge. Interestingly, when we normalized the age of mice at challenge, but infected mice with MCMV at different ages, we observed that the CD8 response to superinfection with WNV was diminished in MCMV infected animals, but was not worsening with the passage of time upon MCMV infection (Fig. 5E and F), Similarly, VACV infection resulted in a transient increase of EM and of KLRG1^+^ fractions, and the only groups of MCMV and VACV infected animals that showed no significant difference to one another in responses to WNV challenge were the groups of animals infected for two months prior to challenge (Fig. 6E and F). Nothwithstanding these minor discrepancies, our results described above and the data from Mekker et al. are highly consistent and demonstrate that MCMV infection results in several phenotypical and functional changes of the immune system that are usually associated with aging.

Surprisingly, we observed that MI does not reflect a progressive expansion of the entire EM pool. In fact, after its initial rapid expansion, the fraction of EM cells in the CD8 pool did not increase further than the levels seen 14 days after infection (Fig. 1C). On the other hand, the CD8 cells recognizing the immunodominant epitope YPHFMPTNL increased in the same mice from approximately 2 to 16% of the CD8 compartment. This implies that after the initial expansion of EM cells, the main change within the CD8 compartment did not involve their further expansion, but rather the replacement of the relatively polyclonal antiviral response by the oligoclonal response against defined epitopes, while the overall magnitude of the response remained unaltered. Therefore, our results raise the question whether the increase of CMV pp65 Ag specific cells observed with advancing age [3], indeed reflects an increase in the population of CMV specific cells, or merely reflects focusing of CMV-specific CD8 responses upon a narrower range of defined immunodominant targets.

Numerous clinical studies showed that CMV seropositivity coincides with poor responses to other viruses [13], poor responses to vaccines [12], or poor life expectancy in the very old [6], [10], [38]. We showed recently that the phenotype and function of CMV-specific T-cells from old and immunosenescent rhesus monkeys are indistinguishable from those in adult and immunocompetent ones [25]. However, the rhesus model did not allow the comparison of CMV infected and uninfected hosts, because essentially all captive rhesus monkeys become naturally infected with CMV early in their life. Therefore, the effects of CMV infection on the aging immune system could only be defined in mouse models of infection, where SPF colonies allow the maintenance of MCMV negative controls throughout their lifetime. To our knowledge, along with the study by Mekker et al., this was the first attempt to establish an experimental model to study this question and the first experimental proof that a persistent herpesvirus infection may lead to an irreversible change in the CD8 pool and impair their ability to respond to emerging infections. While Mekker et al showed clearly that MCMV infection results in a loss of relative and absolute responses to superinfection with unrelated viruses, we showed that the poor responses were exclusive to infection with MCMV and could not be observed in infections with other viruses, such as vaccinia or Herpes simplex, and showed that poor responses are observed in several murine strains and F1 hybrids, arguing that the phenomenon is independent of the mouse gentoype. Mekker et al. showed by adoptive transfer experiments that MCMV infection does not compromise the ability of donor cells to respond to an antigen that they recognize, arguing that MCMV did not impair CD8 function in general, but only the endogenous responses to viruses, whereas we showed that none among the known MCMV immune evasive genes contributes to the suppression of CD8 responses to challenge. Instead we could show a decrease in the recruitment and/or activation of CD8 cells in draining LN upon a viral challenge. Therefore, our results and those from Mekker et al. may have profound implications for our understanding of the immune aging process in a microbiological environment that reflects the real-life exposure of the average individual.

While we focused in this study on the CD8 subset, our data do not exclude the possibility that latent MCMV infection may affect the CD4 T-cell and the B-cell subsets of lymphocytes. Future studies will allow us to elucidate the effect of persisting CMV infections on T-helper subsets and on humoral immune responses, a question particularly important in light of the relevance of these subsets for the efficiency of vaccination strategies.

Based on our data, we propose a model in which the persistent CMV infection causes an ongoing recruitment of EM cells in in lymph nodes, which impairs the ability of the naïve cells to mount responses to unrelated virus. Future studies should elucidate whether and to what extent these effects are due to competition of T-cell populations within the lymph node, or alterations in the migration and/or antigen presenting function of professional APC, or whether there also may be a component of TCR repertoire diversity reduction, for which there is some support (Smithey, M.J. et al., submitted for publication). The newly established balance may offer benefits in terms of stronger innate immune responses [37], yet at the same time diminishes the efficiency of the adaptive branch to target neoantigens. All of these changes, combined with age-related cell-autonomous changes in T-cells [39] or other parts of the immune system (rev in [40]) could contribute to the clinical evidence of poor prognosis and poor CD8 function in very old CMV-positive hosts observed in some studies [6], [9], [41]. Our model should provide a useful tool to elucidate the effects of persistent CMV infection on the immune system and the mechanisms of this interaction.

## Materials and Methods

### Ethics statement

All animal experiments at OHSU were performed according to federal (U.S. Animal Welfare Act) and institutional guidelines, following the Institutional animal care and usage committee (IACUC) requirements, under the protocol #0724. All animal experiments performed at HZI were performed in compliance with the German animal protection law (TierSchG BGBI S. 1105; 25.05.1998) and were approved by the responsible state office (Lower Saxony State Office of Consumer Protection and Food Safety) under permit number 33.9-42502-04-11/0426. The mice were housed and handled in accordance with good animal practice as defined by FELASA and the national animal welfare body GV-SOLAS.

### Virus

MCMV, molecular clones MW97.01 [42] and Δm1-17+144-158 [36] were grown on MEF cells and purified by sucrose cushion ultracentrifugation. WNV, strain 385-99, provided by Dr R.T. Tesh (U. Texas Med. Branch, Galveston, TX), Herpes simplex type 1 virus - strain 17 (HSV-1), syn+, provided by Dr. J. McGeoch (University of Glasgow, Scottland, UK), VACV strain Western reserve (VACV-WR) and MCMV-ieI-Vaccinia Virus (VACV-ie1) [43] were grown and titrated on Vero cells. Influenza PR/8/34 strain (Flu), grown on hen eggs, was purchased from Charles River.

### Mice and mouse experiments

Mice were purchased from NCI intramural breeding colony (Frederick, MD) and housed in barrier BSL2 or BSL3 housing at OHSU West Campus, or purchased from Janvier (France) and housed in barrier BSL2 housing at HZI. The SPF status of experimental mice was confirmed by monitoring sentinels housed on same blue-line racks for the duration of the experiments. Experiments were performed on BALB/c, 129Sv6, (BALB/c×DBA/2) F1, or (DBA/2×C57BL/6N) F1 mice. Unless otherwise indicated, data from (BALB/c×DBA/2) F1 mice are shown, and this cohort was additionally controlled on a cage basis for murine Norovirus by serology during the course of the experiment (all CMV and mock-infected mice were negative). Mice were intraperitoneally (i.p.) infected with 10^5^ PFU of MCMV or 10^6^ PFU of VACV. Challenge assays were performed with 100 PFU of WNV or 10^6^PFU of HSV-1 applied i.p., or intranasally with 300 EID^50^ of the Flu virus.

### Peptide stimulation and screening

We stimulated blood leukocytes corresponding to 100 µl blood with 10^−6^ M concentration of peptides in RPMI (5% FBS, 5 µg/ml Brefeldin A) for 6 h at 37°C. We used influenza peptides NP_366–374_ (peptide sequence ASNENMETM) or PA_224–233_ (SSLENFRAYV), the HSV-1 gB peptide SSIEFARL, the CMV peptide YPHFMPTNL (IE-1 derived) or the WNV peptides identified by screening (see below). Negative controls consisted of cells that were incubated in the same conditions in the presence of an ovablumin peptide (OVA, SIINFEKL). Upon stimulation, the cells were washed and subjected to surface staining and intracellular cytokine staining (ICCS). To identify H-2^d^ restricted immunodominant WNV peptides, we screened a library of 684 peptides (15-mers overlapping by 10 peptides) spanning the entire WNV genome and identified two immunodominant 15mers that induced cytokine responses upon in vitro restimulation of splenocytes from WNV infected mice. Two optimal H2-K^d^-restricted restricted nonamers (KYVDYMSSL and GYISTKVEL) were predicted using a public web-service for epitope prediction [44] (available at www.syfpeithi.de) and their ability to induce in vitro cytokine responses was experimentally validated by in vitro restimulation of splenocytes and ICCS.

### Flow cytometry (FCM)

Erythrocytes were lysed by brief (5 s) osmotic shock in dH_2_O, and blood leukocytes were stained at +4C for 30′ with fluorophore-conjugated monoclonal antibodies. We used as our standard an 8-color panel anti(α) CD3-APC-Alexa750 (eBioscience, San Diego, CA), αCD4-Pacific Blue (BD, San Jose, CA), αCD8-Percp-Cy5.5 (BD, San Jose, CA), αCD11a-PE-Cy7 (eBioscience, San Diego, CA), αCD44-Alexa700, αCD62L-PE-Texas Red (Invitrogen, Carlsbad, CA), αCD127-PE, and αKLRG1-biotin followed by Streptavidin-Qdot525. In some experiments, we also used αLy6C-FITC (eBioscience, San Diego, CA), αCD27-APC (Biolegend) or YPHFMPTNL-L^d^ tetramers coupled to APC (NIH tetramer core facility). For ICCS, cells were washed after the surface staining with the 8-color phenotype panel described above and then fixed for 5′ in 100 µl of IC-fixation buffer (eBioscience, San Diego, CA), incubated for additional 5′ in permeabilization buffer (eBioscience, San Diego, CA), washed twice and stained with anti-IFNγ-APC (BD, San Jose, CA) for 1 h. Upon two washes in permeabilization buffer, cells were acquired in an LSR2 flow-cytometer (BD, San Jose, CA) and analysis was performed by FlowJo software (TreeStar, Ashland, OR).

### Counting the cell populations in absolute terms

In select bleeds, an aliquot (100 µl) of whole blood was directly analyzed in an AC•T 5diff hematologic analyzer (Beckman-Coulter) or CD8^+^CD4^−^ leukocytes from a defined fraction of spleen or lymph-node homogenates were counted in an Accuri C6 flow-cytometer (BD) to define total lymphocyte counts per sample.

### Spectratyping CDR3 length polymorphism (spectratype) analysis

PCR for each of the 22 variant (V) regions of the TCR® chain (Vβ) was performed essentially as described [45], with minor modifications. We isolated CD8^+^, CD3^+^, CD4^−^ splenocytes from 129Sv6 mice infected for 14 months with MCMV or their uninfected littermates using a FACS-ARIA II sorter (BD Biosciences, San Jose, CA), isolated their mRNA and subjected it to a panel of 22 reverse transcriptase (RT) PCR reactions specific for each Vβdomain. PCR products were subjected to electrophoresis on a polyacrylamide gel (PAGE), and analyzed by densitometry in an ABI 3100 sequencer apparatus. V regions of different TCR clones differ in their sequence, but also in size, due to random addition and subtraction of nucleotides during somatic recombination. Thus, PCR of V TCR regions, results in products of varying lengths, where products with the median length of about 10 aa (30 nt) are the most abundant, followed in a descending order by clones that are progressively shorter or longer than this median length. PCR followed by PAGE and densitometry, allows the differentiation of polyclonal cell populations, with bell-shaped distribution of PCR products in PAGE densitometry, from the oligoclonal populations, where reduced diversity does not allow the formation of a Gaussian curve.

### Statistical analysis

Statistical analysis was performed with GraphPad Prism software.

## Supporting Information

Figure S1 Representative gating of Ly6C^+/−^ populations of CD62L^−^, CD8^+^ lymphocytes in mice infected with mouse CMV, VACV or mock infected controls. Cells were gated on the EM gate (see Figure 1 B) and then analyzed for Ly6C expression.
(PPT)

Figure S2 Representative progressive gating of blood CD8 lymphocytes used to define naïve cells on a CD11a^−^CD44^−^CD62L^+^CD127^+^ gate (see Fig. 3C).
(PPT)

Figure S3 Representative gating strategy used to define naïve, CM and EM CD8 T-cells in the blood, spleen and LN of MCMV infected mice. Cells were gated first on a CD11a^−^CD44^−^ or a CD11a^+^CD44^+^ gate, upon which they were gated on a CD62L^+^CD27^+^ gate, allowing us to define the CD11a^−^CD44^−^CD27^+^CD62L^+^ (naïve) or the CD11a^+^CD44^+^CD27^+^CD62L^+^ (CM) cells. CD62L^−^ cells were collected from all gates and combined to define the percentage of EM cells. We replaced CD127 with CD27 for the purpose of this gating, because all CD8 cells from LN were CD127^+^, and because they did not clearly separate in distinct CD62L^+^ and CD62L^−^ subsets. Replacing CD127 with CD27 allowed us to identify subsets of LN cells lacking two receptors normally found on CM and naïve but not on effector cells, and it allowed us to identify the boundaries between the positive and negative CD62L fractions.
(PPT)

Figure S4 C57BL/6 mice were infected with 10^6^ PFU of HSV-1 at 4 months of age. Littermate control mice were allowed to age in the absence of any infection. At 18 months of age both groups were i.p. infected with 100 PFU of WNV and the percentage of cells specific for the immunodominant peptide SSVWNATTA (Brien et al. Eur J Immunol. 2007 Jul;37(7):1855–63) in the CD8 pool was determined by pMHC staining and flow cytometry at 7 days post infection.
(PPT)

Figure S5 129Sv6xBALB/c mice were infected with indicated viruses at 6, 12, 16 or 20 months of age and challenged with WNV at 22 months of age. Mice were bled 7 days post infection and blood leukocytes were stimulated with anti-CD3 antibodies for 6 h in the presence of brefeldin A, upon which the CD8^+^ cells were stained for intracellular IFNγ expression and acquired in an LSR2 cytometer. % of IFNγ^+^ cells in the CD8 pool are shown in the y axis. Symbols show individual mice, horizontal bars are means, cells were compared by ANOVA, followed by Bonferroni post analysis for the indicated groups and ns denotes p values above 0.1.
(PPT)

Figure S6 Absolute counts of CD8 cells in medastinal LN of MCMV or VACV infected BALB/Uc mice were analyzed at 6 months post infection. Cell counts in individual mice are displayed, horizontal lines indicate medians.
(PPT)
