# Supplementary figures and images for: Cytomegalovirus Infection Impairs Immune Responses and Accentuates T-cell Pool Changes Observed in Mice with Aging
Source: PLoS Pathog. 2012 Aug 16;8(8):e1002849. doi: 10.1371/journal.ppat.1002849 (PMC3420928; doi:10.1371/journal.ppat.1002849)

## Slide 1
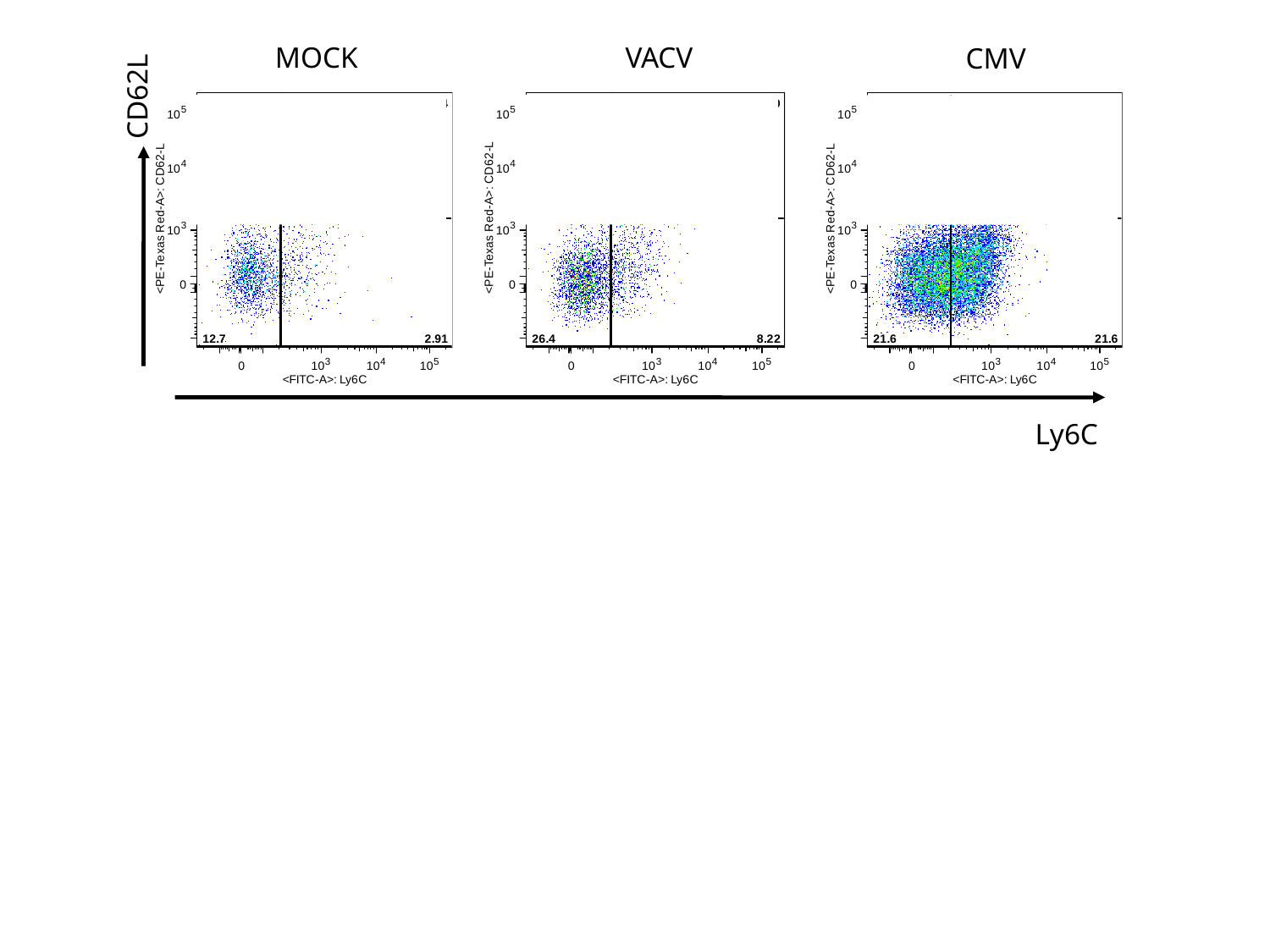

MOCK
VACV
CMV
CD62L
Ly6C

Supplement: Figure S1 — Representative gating of Ly6C+/− populations of CD62L−, CD8+ lymphocytes in mice infected with mouse CMV, VACV or mock infected controls. Cells were gated on the EM gate (see Figure 1 B) and then analyzed for Ly6C expression. (PPT) [file ppat.1002849.s001.ppt]

## Slide 1
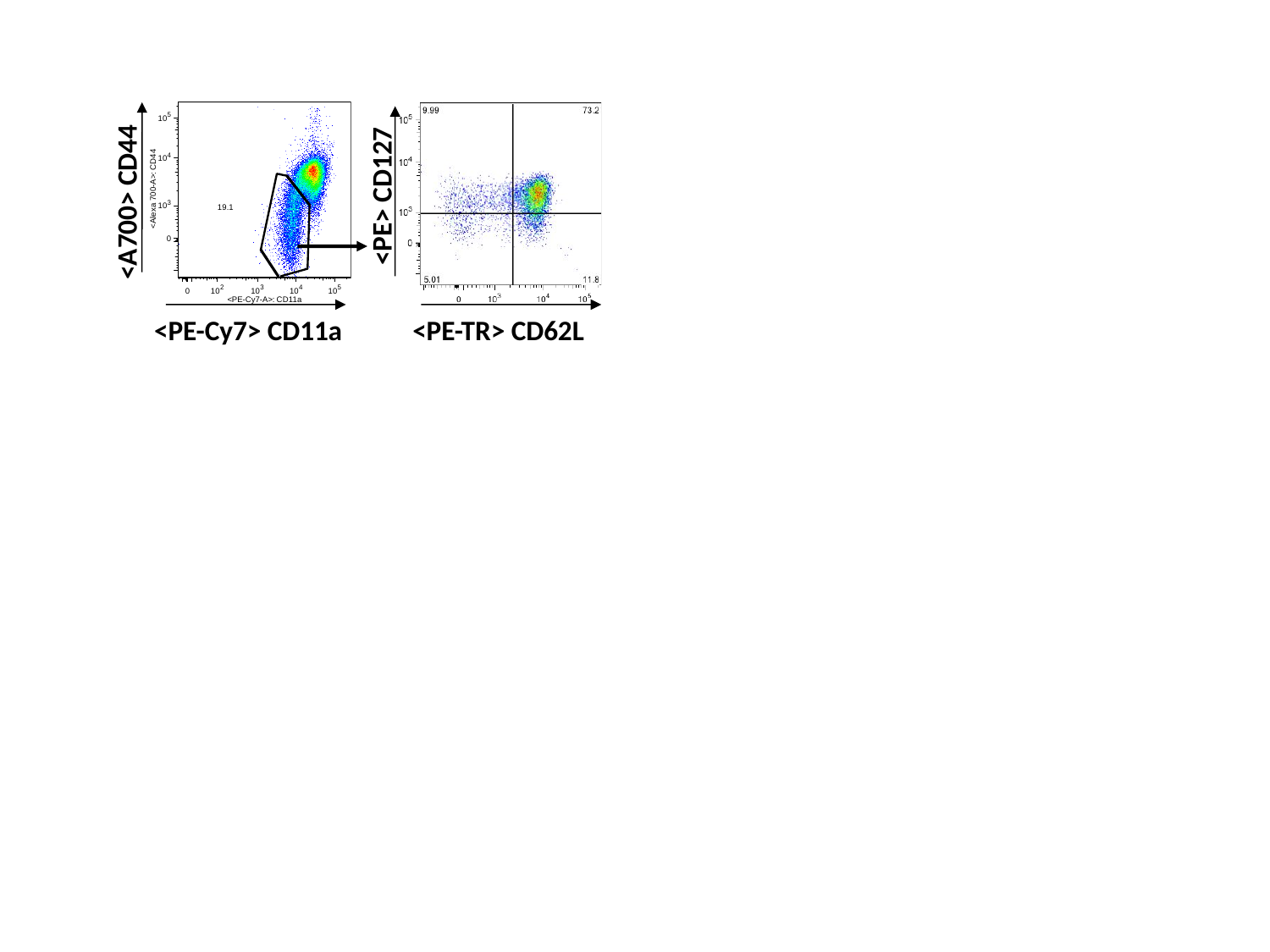

<PE> CD127
<A700> CD44
<PE-Cy7> CD11a
<PE-TR> CD62L

Supplement: Figure S2 — Representative progressive gating of blood CD8 lymphocytes used to define naïve cells on a CD11a−CD44−CD62L+CD127+ gate (see Fig. 3C). (PPT) [file ppat.1002849.s002.ppt]

## Slide 1
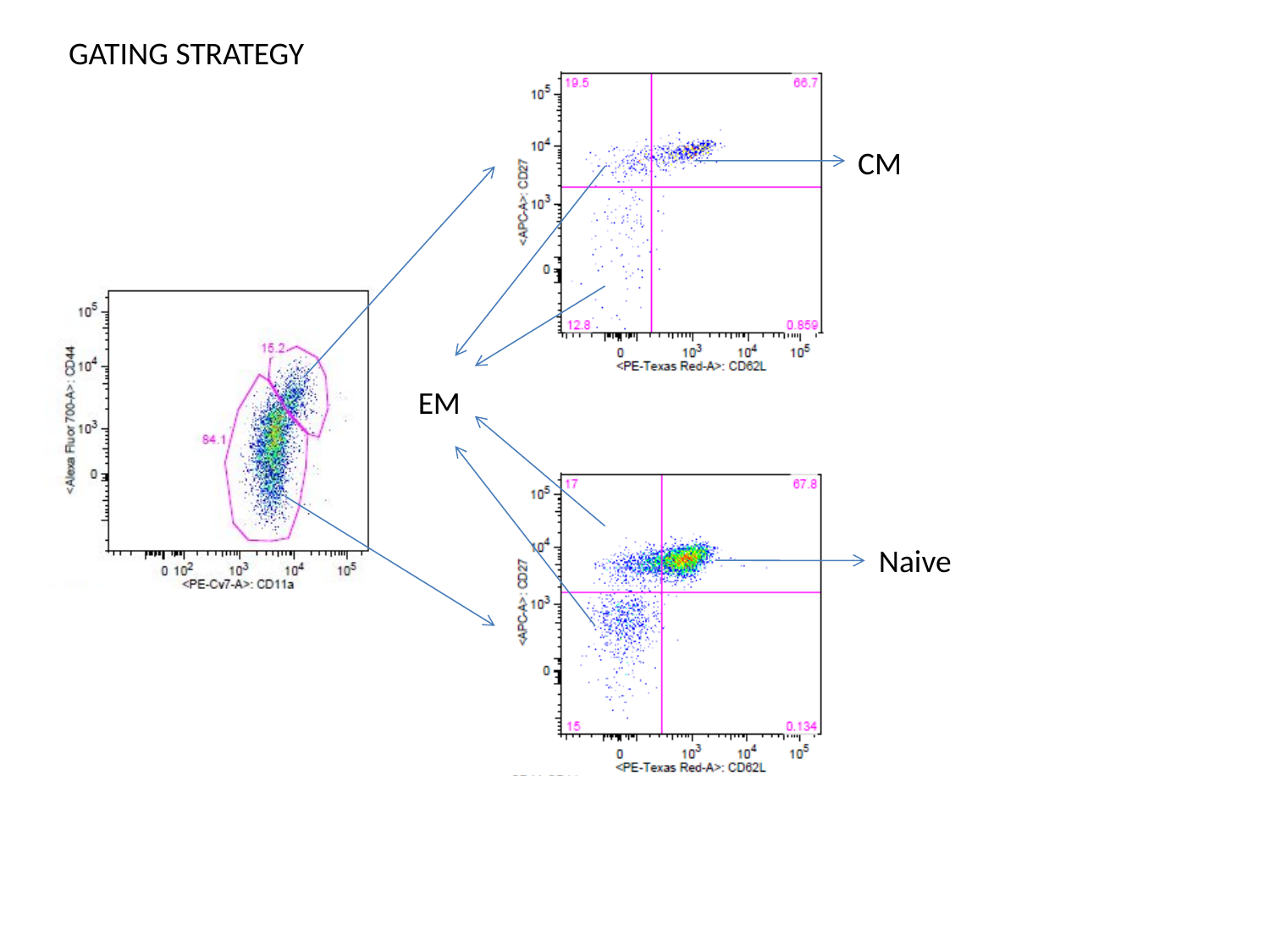

GATING STRATEGY
CM
EM
Naive

Supplement: Figure S3 — Representative gating strategy used to define naïve, CM and EM CD8 T-cells in the blood, spleen and LN of MCMV infected mice. Cells were gated first on a CD11a−CD44− or a CD11a+CD44+ gate, upon which they were gated on a CD62L+CD27+ gate, allowing us to define the CD11a−CD44−CD27+CD62L+ (naïve) or the CD11a+CD44+CD27+CD62L+ (CM) cells. CD62L− cells were collected from all gates and combined to define the percentage of EM cells. We replaced CD127 with CD27 for the purpose of this gating, because all CD8 cells from LN were CD127+, and because they did not clearly separate in distinct CD62L+ and CD62L− subsets. Replacing CD127 with CD27 allowed us to identify subsets of LN cells lacking two receptors normally found on CM and naïve but not on effector cells, and it allowed us to identify the boundaries between the positive and negative CD62L fractions. (PPT) [file ppat.1002849.s003.ppt]

## Slide 1
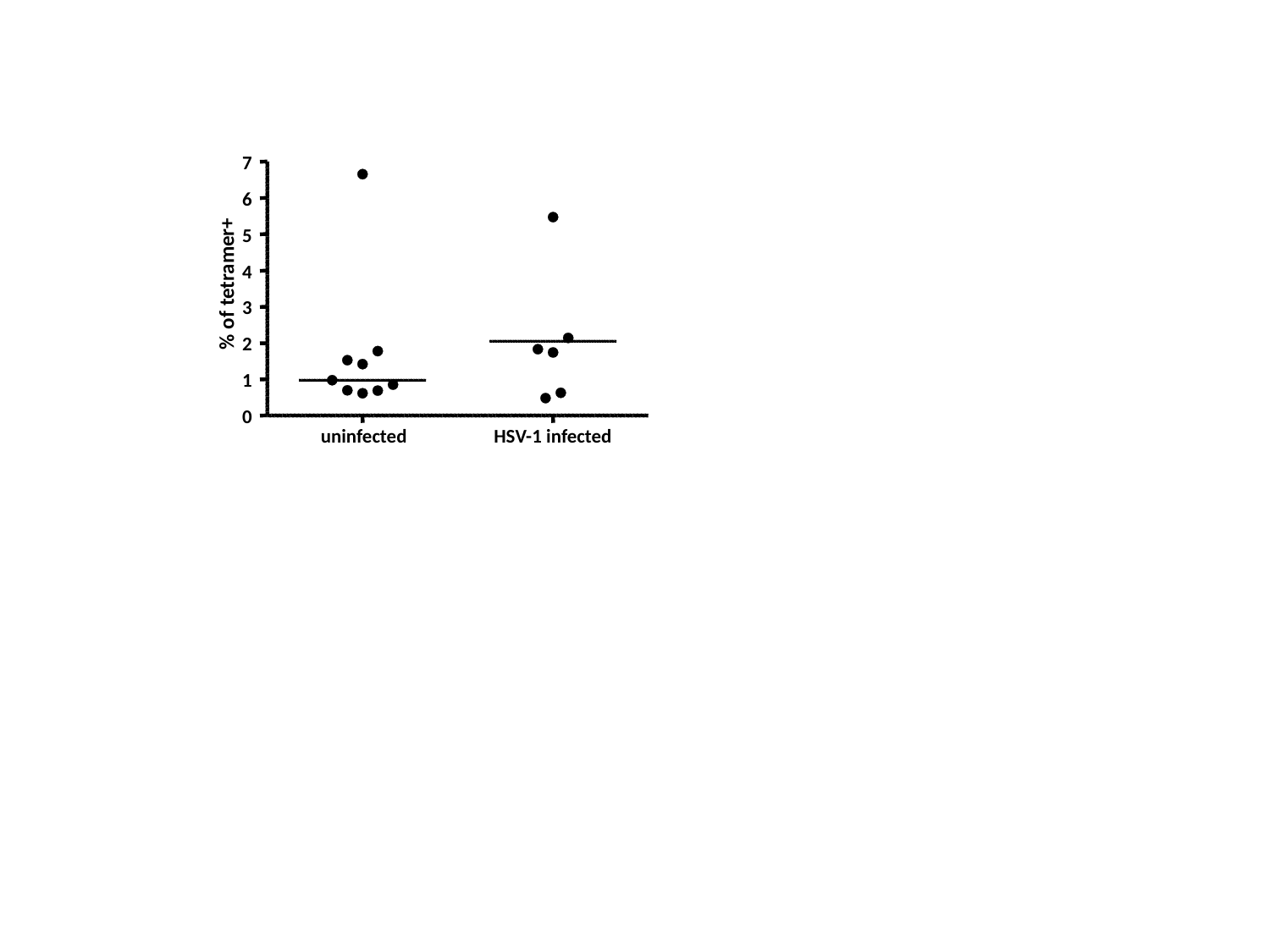

7
6
5
4
3
2
1
0
uninfected
HSV-1 infected
% of tetramer+

Supplement: Figure S4 — C57BL/6 mice were infected with 106 PFU of HSV-1 at 4 months of age. Littermate control mice were allowed to age in the absence of any infection. At 18 months of age both groups were i.p. infected with 100 PFU of WNV and the percentage of cells specific for the immunodominant peptide SSVWNATTA (Brien et al. Eur J Immunol. 2007 Jul;37(7):1855–63) in the CD8 pool was determined by pMHC staining and flow cytometry at 7 days post infection. (PPT) [file ppat.1002849.s004.ppt]

## Slide 1
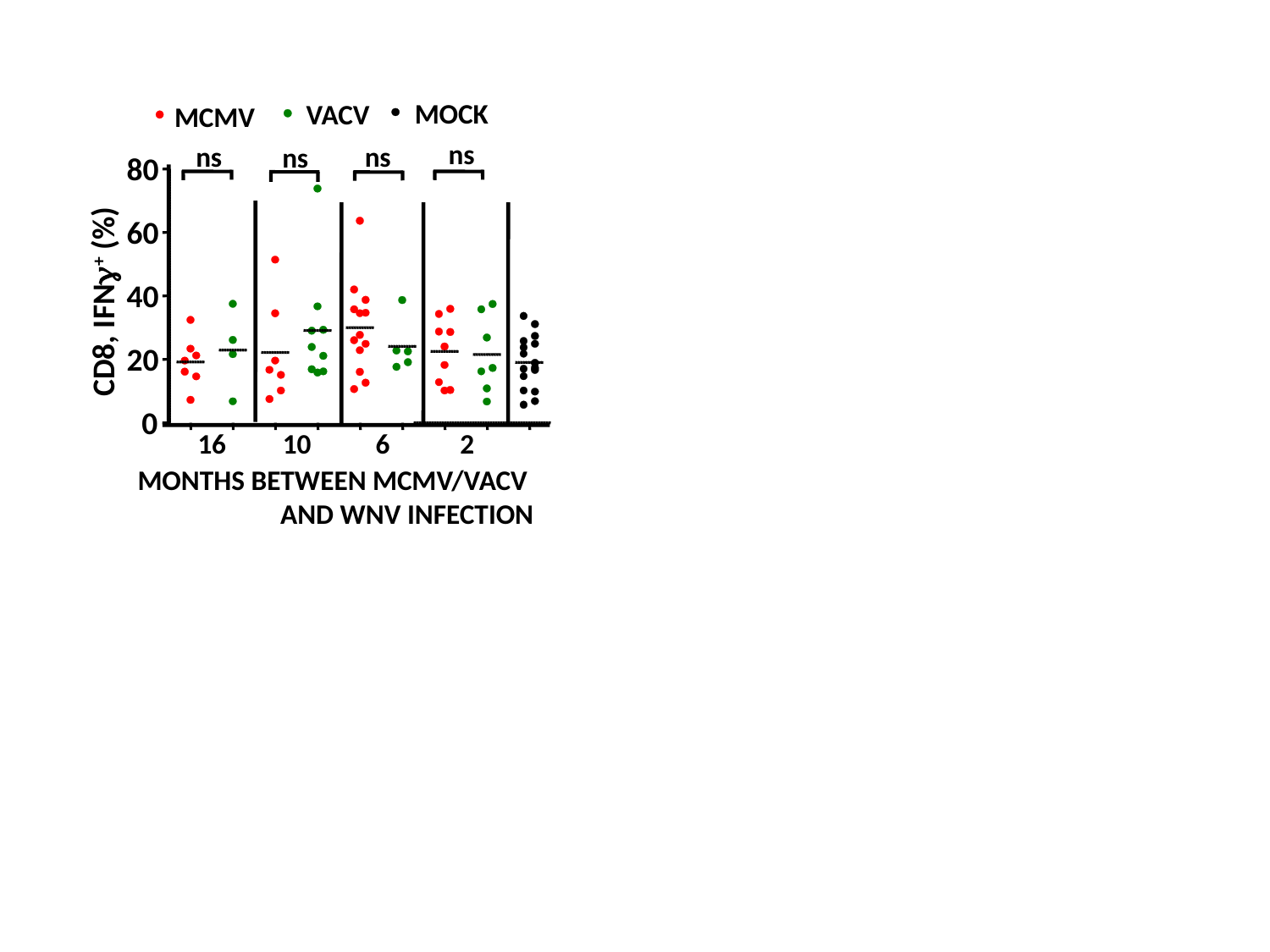

MOCK
VACV
MCMV
ns
ns
ns
ns
80
60
40
20
0
CD8, IFNγ+ (%)
16
10
6
2
MONTHS BETWEEN MCMV/VACV
AND WNV INFECTION

Supplement: Figure S5 — 129Sv6xBALB/c mice were infected with indicated viruses at 6, 12, 16 or 20 months of age and challenged with WNV at 22 months of age. Mice were bled 7 days post infection and blood leukocytes were stimulated with anti-CD3 antibodies for 6 h in the presence of brefeldin A, upon which the CD8+ cells were stained for intracellular IFNγ expression and acquired in an LSR2 cytometer. % of IFNγ+ cells in the CD8 pool are shown in the y axis. Symbols show individual mice, horizontal bars are means, cells were compared by ANOVA, followed by Bonferroni post analysis for the indicated groups and ns denotes p values above 0.1. (PPT) [file ppat.1002849.s005.ppt]

## Slide 1
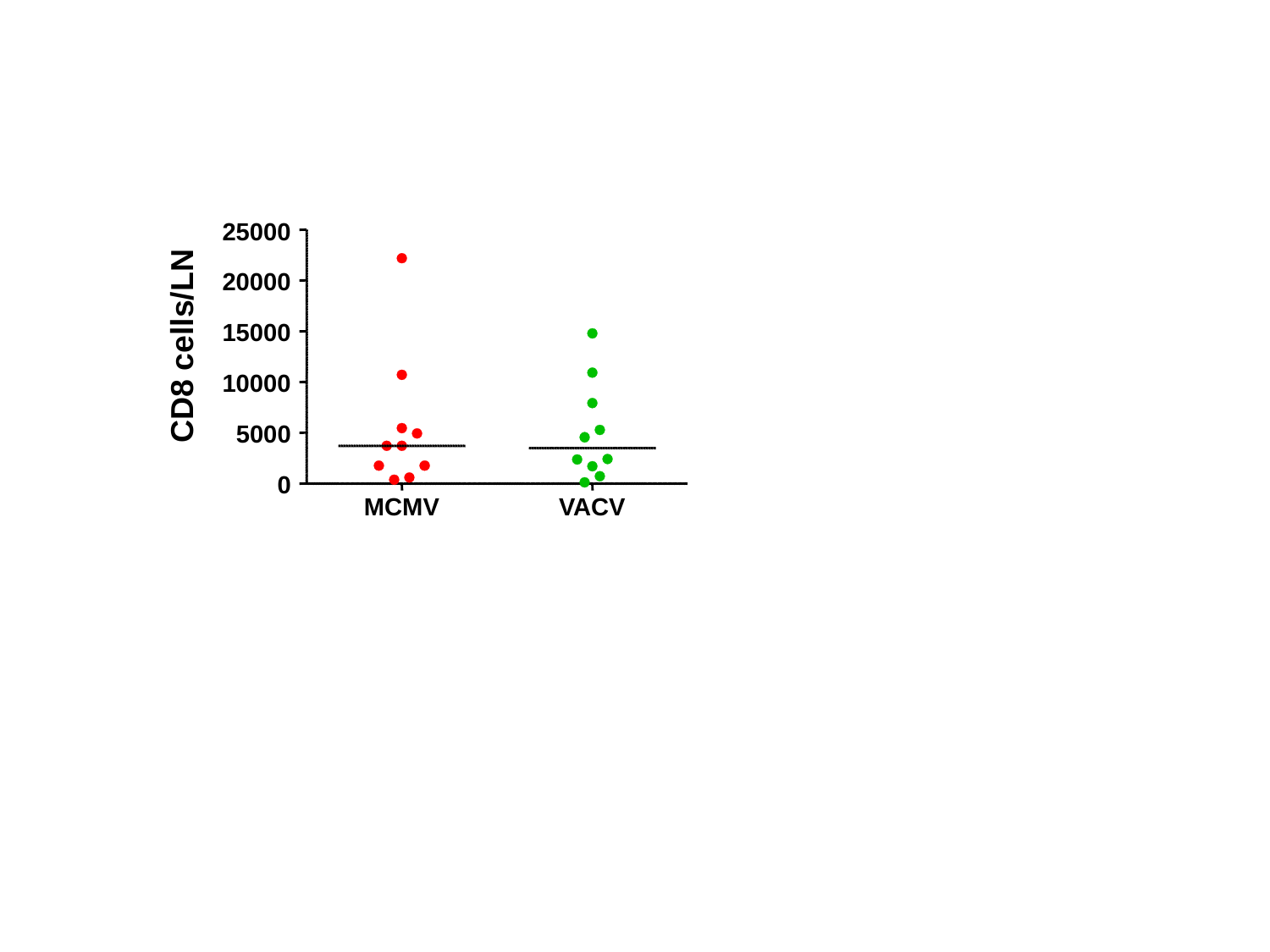

MCMV
VACV
25000
20000
15000
CD8 cells/LN
10000
5000
0

Supplement: Figure S6 — Absolute counts of CD8 cells in medastinal LN of MCMV or VACV infected BALB/Uc mice were analyzed at 6 months post infection. Cell counts in individual mice are displayed, horizontal lines indicate medians. (PPT) [file ppat.1002849.s006.ppt]
